# Enhancing Rheology and Wettability of Drilling Fluids at Ultra-Low Temperatures Using a Novel Amide Material

**DOI:** 10.3390/gels11090687

**Published:** 2025-08-28

**Authors:** Ning Huang, Jinsheng Sun, Jingping Liu, Kaihe Lv, Xuefei Deng, Taifeng Zhang, Yuanwei Sun, Han Yan, Delin Hou

**Affiliations:** 1 School of Petroleum Engineering, China University of Petroleum (East China), Qingdao 266580, China; 2 CNPC Engineering Technology R & D Company Limited, Beijing 102206, China

**Keywords:** Antarctic drilling fluid, ultra-low temperature, rheology, weak gel structure, amide material

## Abstract

The ice sheet and subglacial geological environment in Antarctica have become the focus of scientific exploration. The development of Antarctic drilling technology will serve as a crucial safeguard for scientific exploration. However, the extremely ultra-low temperatures and intricate geological conditions present substantial obstacles for drilling operations in Antarctica, and the existing drilling fluid technology cannot satisfy the requirements of efficient and safe drilling. To ameliorate the wettability and rheology of ultra-low-temperature drilling fluids, a new amide material (HAS) was prepared using dodecylamine polyoxyethylene ether, azelaic acid, and *N*-ethylethylenediamine as raw materials. Experiments using infrared spectroscopy, nuclear magnetic hydrogen spectroscopy, and contact angle indicated that the target product was successfully synthesized. Performance evaluation showed that 2% HAS could achieve a yield point of 2.5 Pa for drilling fluid at −55 °C, and it also gave the fluid superior shear-thinning characteristics and a large thixotropic loop area. This indicated that HAS significantly enhanced the rheological properties of the drilling fluid, ensuring that it can carry cuttings and ice debris. In addition, 2% HAS could also increase the colloidal rate from 8% to more than 76% at −55 °C in different base oils. Meanwhile, the colloid rate was maintained above 92.4% when the density was 0.92~0.95 g/cm^3^. Mechanism studies showed that HAS increased the zeta potential and decreased the particle size of organoclay. At the same time, it changed the organoclay state from a clustered state to a uniformly dispersed state, and the particle size decreased. It was found that HAS formed a weak gel grid structure through interactions between polar groups, such as amide and imino groups with organoclays particles, thus improving the rheology and wettability of drilling fluid. In addition, HAS is an environmentally friendly high-performance material.

## 1. Introduction

The strata in Antarctica are mainly composed of ice sheets, ice rock interlayers, and subglacial bedrock. Obtaining ice cores and rock cores is of great significance for understanding geological structures, revealing the evolutionary history of ice sheets and assessing future climate change. Developing Antarctic drilling technology is an important necessity for obtaining the above information. However, in the drilling process in Antarctica, extreme low-temperature conditions and complex geological structures lead to great challenges in drilling operations, mainly including low-temperature resistance of drilling equipment, low drilling efficiency, and other problems [1,2,3,4,5]. Drilling fluid is a significant component of the drilling process, and Antarctic drilling fluids mainly suffer from poor low-temperature rheology, inadequate wettability, and limited environmental compatibility [6]. These problems seriously hinder the smooth progress of drilling operations. Therefore, improving drilling fluid technology will be very necessary and indispensable for safe and efficient drilling in Antarctica.

Excellent wettability is crucial to ensure that the rheological and filtration performance of drilling fluid is adequate. In conventional drilling fluid research, scholars have often achieved outstanding results by developing wetting agents to improve the lipophilic properties of barite and organoclays, which ultimately improves the wettability of drilling fluids [7,8,9]. Murtaza et al. addressed the issue of barite settling in drilling fluids by using non-ionic wetting agents, such as alcohol alkoxylates, to effectively improve wettability [10]. Li et al. employed sodium dodecylbenzenesulphonate as an anionic wetting agent to enhance the suspension of barite, which significantly improved drilling fluid properties [11]. Sui et al. optimized key treatment agents, such as organoclays, emulsifiers, and anionic wetting agents, to construct a drilling fluid system. Among them, a 0.4% wetting agent allowed the barite volume to still reach 90 mL (standing for 170 min), improving the barite lipophilicity and drilling fluid rheology [12]. Paswan et al. synthesized a material (SMES) from sunflower oil, lowering interfacial tension by 77.15 mN/m and boosting the contact angle by 49.8°, which improved the emulsion stability and oil-wetting capability [13]. In researching a rheological modifier for oil-based drilling fluid, Ma et al. [14] synthesized a polymer rheological modifier (PRM) from methacrylate, methylstyrene, octadecyl acrylate, and dimethyl azodiisobutyrate. It was found that 2% PRM could increase the yield point of drilling fluid from 0 Pa to 5 Pa at 180 °C, and it had good cuttings carrying performance. Mahmoud et al. prepared a new rheological modifier (Claytone img 400) from montmorillonite, quaternary ammonium salt, and other raw materials, which increased the ratio of the yield point and plastic viscosity from 1.12 Pa to 1.19 Pa and improved the yield point by 38%. In addition, it also had good filtration reduction performance [15]. He et al. synthesized an amphiphilic multiblock polymer through the amidation reaction of polyfatty acid and polyether amine, which could make the yield point of drilling fluid exceed 10 Pa and G’ exceed 7 Pa after aging at 220 °C for 24 h, significantly enhancing the rheological properties of drilling fluid [16]. However, the above research on wetting agents and rheological modifiers was only conducted for conventional formation drilling. There is currently almost no relevant reports on the research of wetting agents and rheological modifiers for Antarctic drilling fluids. Therefore, it is urgent to develop new drilling fluid materials suitable for the Antarctic region to promote efficient drilling.

Organoclay is the main dispersed phase of Antarctic drilling fluids, with its low-temperature wettability being a decisive factor for rheological performance. Organoclay has been applied to optimize the rheological properties of drilling fluids. Nevertheless, studies have shown that it has poor ultra-low-temperature wettability, severely affecting drilling fluids’ rheology. Therefore, to improve the wettability and rheology of ultra-low-temperature drilling fluids, it is necessary to prepare a material that can significantly enhance the performance of Antarctic drilling fluids.

In this study, an environmentally friendly material (HAS) was prepared from dodecylamine polyoxyethylene ether, azelaic acid, and *N*-ethyl ethylenediamine, which obviously enhanced the wettability and rheology of Antarctic drilling fluid. The HAS structure was analyzed by infrared spectroscopy, nuclear magnetic hydrogen spectroscopy, and contact angle experiments. Then, the wetting and rheological performance of HAS at ultra-low temperatures and its environmental friendliness were also assessed. Ultimately, the action mechanism of HAS was deeply explored through methods such as FBRM, cryo-SEM, and zeta potential. This study establishes the foundation for the development of Antarctic drilling fluid and provides effective support for scientific exploration in Antarctica.

## 2. Results and Discussion

### 2.1. Structural Characterization

#### 2.1.1. Infrared Spectrum

**Figure 1:**
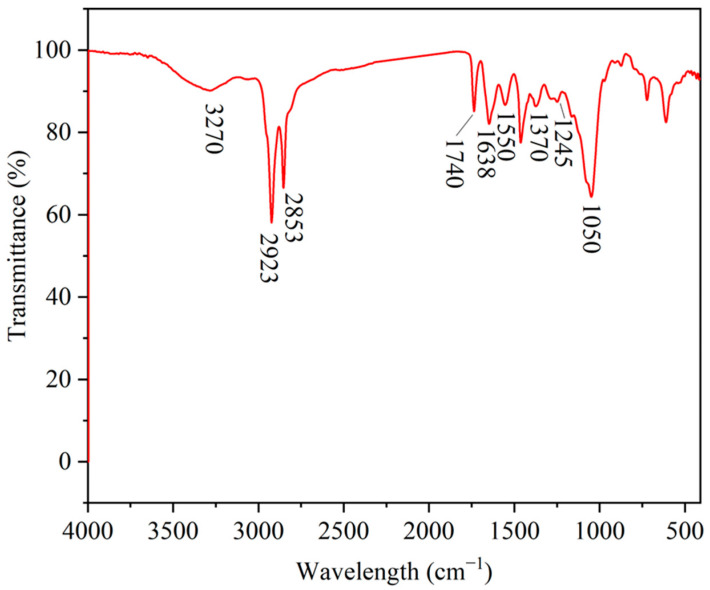
Infrared spectrogram of HAS.

The IR spectra of HAS are shown in Figure 1. The peak near 3270 cm^−1^ is the stretching vibration of N-H in the amide group. The peaks near 2923 cm^−1^ and 2853 cm^−1^ correspond to the antisymmetric and symmetric stretching vibration of C-H, respectively. The peak near 1740 cm^−1^ corresponds to the C=O stretching vibration of the ester group. The corresponding peak near 1638 cm^−1^ is the stretching vibration peak of C=O in the amide group. The corresponding peak near 1550 cm^−1^ is the bending vibration peak of N-H, and the peak near 1370 cm^−1^ corresponds to the bending vibration peak of C-H. The peak near 1245 cm^−1^ corresponds to the stretching vibration peak of C-C. The peak near 1050 cm^−1^ corresponds to the stretching vibration peak of C-N.

#### 2.1.2. Nuclear Magnetic Hydrogen Spectrum

**Figure 2:**
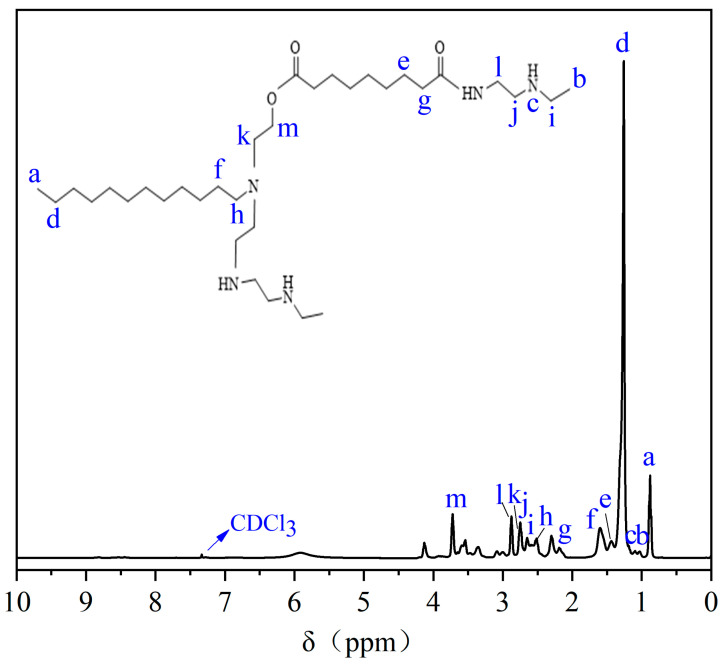
NMR hydrogen spectrum of HAS.

The ^1^H NMR results of HAS in CDCl_3_ are shown in Figure 2. Peak a (0.88 ppm) is the proton peak of -CH_3_ at the end of dodecylamine polyoxyethylene ether. Peak b (1.11 ppm) is the proton peak of -CH_3_ in the *N*-ethyl ethylenediamine, and peak c (1.15 ppm) is the proton peak of -NH in *N*-ethyl ethylenediamine. Peak d (1.26 ppm) is the proton peak of -CH_2_ in the -CH_3_CH_2_ for dodecylamine polyoxyethylene ether, and peak f (1.60 ppm) is the peak of -CH_2_ in the -CH_2_CH_2_N for dodecylamine polyoxyethylene ether. Peak e (1.40 ppm) is the proton peak of -CH_2_ for nonanedioic acid. Peak g (2.19 ppm) is the proton peak of -CH_2_ in the structure of -CH_2_COOH for azelaic acid. Peak h (2.50 ppm) is the proton peak of -CH_2_ in the structure of -CH_2_N for dodecylamine polyoxyethylene ether. Peak i (2.66 ppm) is the proton peak of -CH_2_ in the structure of -CH_3_CH_2_ for *N*-ethyl ethylenediamine, and peak j (2.71 ppm) is the proton peak of -CH_2_ in the structure of -CH_3_CH_2_NHCH_2_ for *N*-ethyl ethylenediamine. Peak k (2.74 ppm) is the proton peak of -CH_2_ in the structure of -CH_2_CH_2_OH for dodecylamine polyoxyethylene ether. Peak l (2.81 ppm) is the proton peak of -CH_2_ in the structure of -CH_2_NH for *N*-ethyl ethylenediamine. Peak m (3.68 ppm) is the proton peak of -CH_2_ in the structure of -CH_2_OH in dodecylamino polyoxyethylene ether, and the peak (7.26 ppm) is the solvent peak of CDCl_3_. ^1^H NMR outcomes indicated the successful preparation of HAS.

### 2.2. Performance Evaluation

#### 2.2.1. Rheological Properties of HAS

Rheology is the key characteristic for drilling fluids. The rheological performance of HAS was explored.

##### The Impact of HAS on the Viscosity and Yield Point for Drilling Fluid

**Figure 3:**
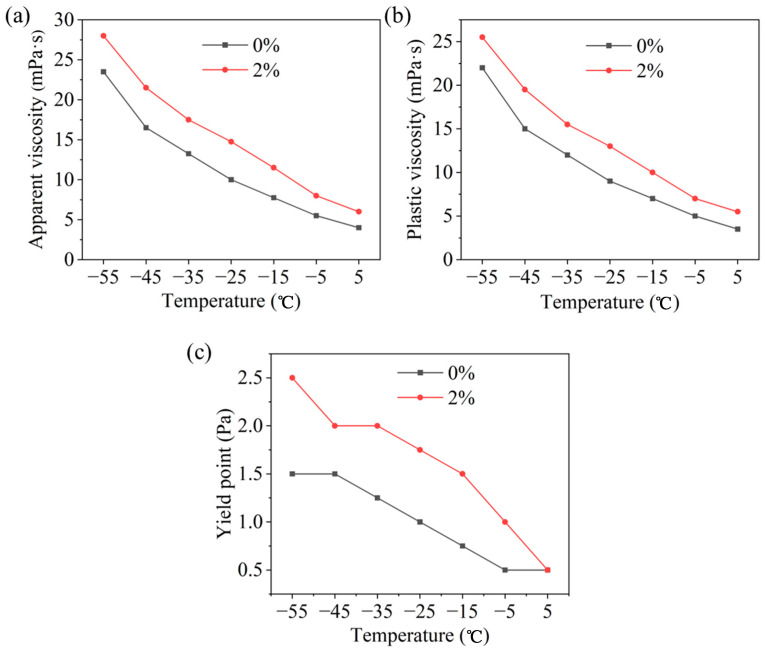
Rheological properties of HAS in drilling fluid ((**a**) apparent viscosity; (**b**) plastic viscosity; (**c**) yield point).

From Figure 3, after adding 2% HAS, the viscosity increased at different temperatures, but the increase was small, with a plastic viscosity of only 25.5 mPa·s at −55 °C. Concurrently, the yield point increased from 1.5 Pa to 2.5 Pa at −55 °C, indicating that HAS significantly enhanced the rheological performance and provided a guarantee for the cuttings carrying capability of drilling fluid.

##### The Impact of HAS on the Shear Dilution of Drilling Fluid

**Figure 4:**
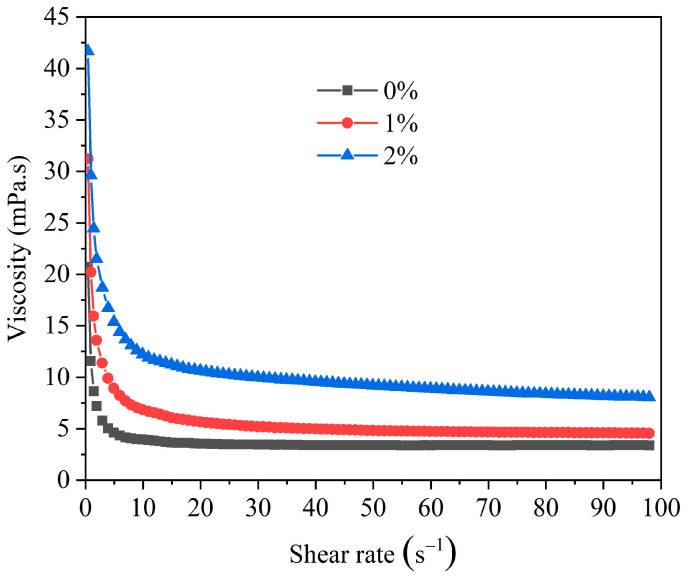
The effect of HAS concentration on the shear dilution for drilling fluid.

Shear dilution stands as one of the vital characteristics for evaluating the rheology for drilling fluids. It directly determines drilling efficiency and safety. Excellent shear dilution performance can endow drilling fluids with outstanding cuttings carrying properties. The influence of HAS on shear dilution was investigated (Figure 4).

When HAS was added, the viscosity of drilling fluid increased at different shear rates. Simultaneously, the viscosity exhibited a sharp decline with increasing shear rate. In addition, at low shear rates, the viscosity was significantly higher than that of the drilling fluid without HAS, substantially improving its capacity to suspend cuttings and ice particles. The above results indicated that HAS could endow drilling fluids with excellent shear dilution properties.

##### The Impact of HAS on the Thixotropy

**Figure 5:**
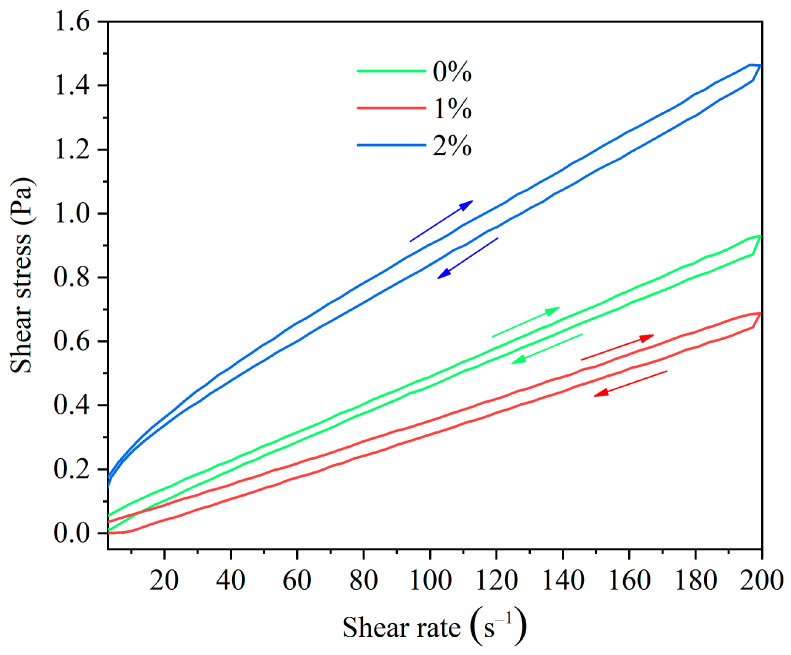
Effect of HAS concentration on the thixotropic loop of drilling fluid.

Thixotropy is one of the important rheological parameters of drilling fluids. The thixotropic loop is one of the most commonly methods to analyze thixotropy, and its area represents the amount of energy needed to disrupt the microstructure of drilling fluids. If the area is larger, the required energy is greater, which indicates that the structure strength of drilling fluids is greater. As the HAS dosage increased, the area gradually enlarged (Figure 5). This indicated that HAS enhanced the drilling fluid mesh structure, thereby significantly enhancing the capacity to carry cuttings and ice debris.

##### The Effect of HAS on the Modulus

**Figure 6:**
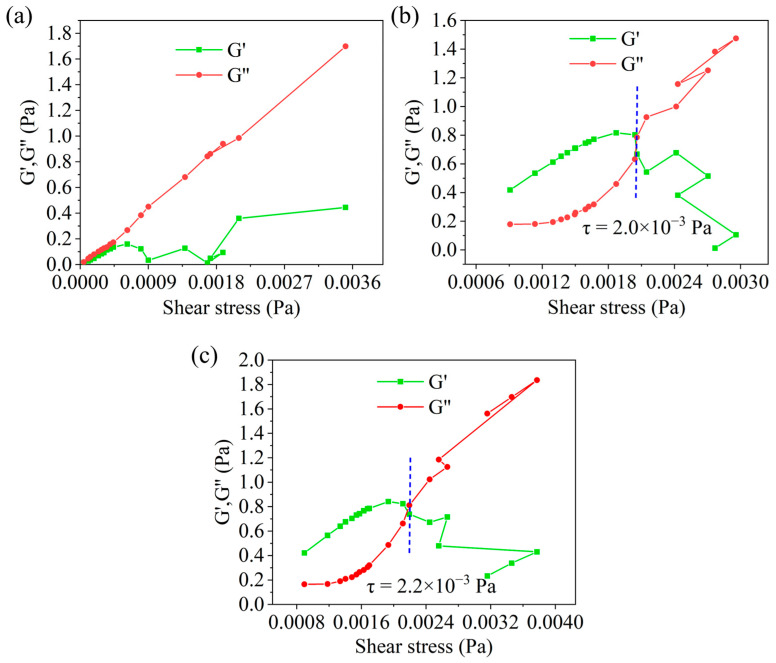
Effect of HAS concentration on drilling fluid modulus ((**a**) 0%; (**b**) 1%; (**c**) 2%).

The modulus includes the elastic modulus (G′) and the viscous modulus (G″). Their relative magnitudes can indicate the structure strength of drilling fluids. From Figure 6a, when no HAS was added, the modulus was primarily dominated by the viscous modulus (G″), indicating that the drilling fluid behaved mainly as a viscous fluid. However, for the drilling fluid with 1% HAS, when the shear stress was below 2.0 × 10^−3^ Pa (Figure 6b), the elastic modulus exceeded the viscous modulus, suggesting that it behaved as an elastic fluid with a higher structural strength. Furthermore, 2% HAS further increased the shear stress value dominated by elastic modulus (Figure 6c). It was analyzed that the polar groups in HAS formed a network structure with hydroxyl groups in organoclay through hydrogen bonding, which enhanced the internal structural forces of the drilling fluid, ultimately leading to elastic fluid behavior.

##### The Analysis of Rheological Model

**Figure 7:**
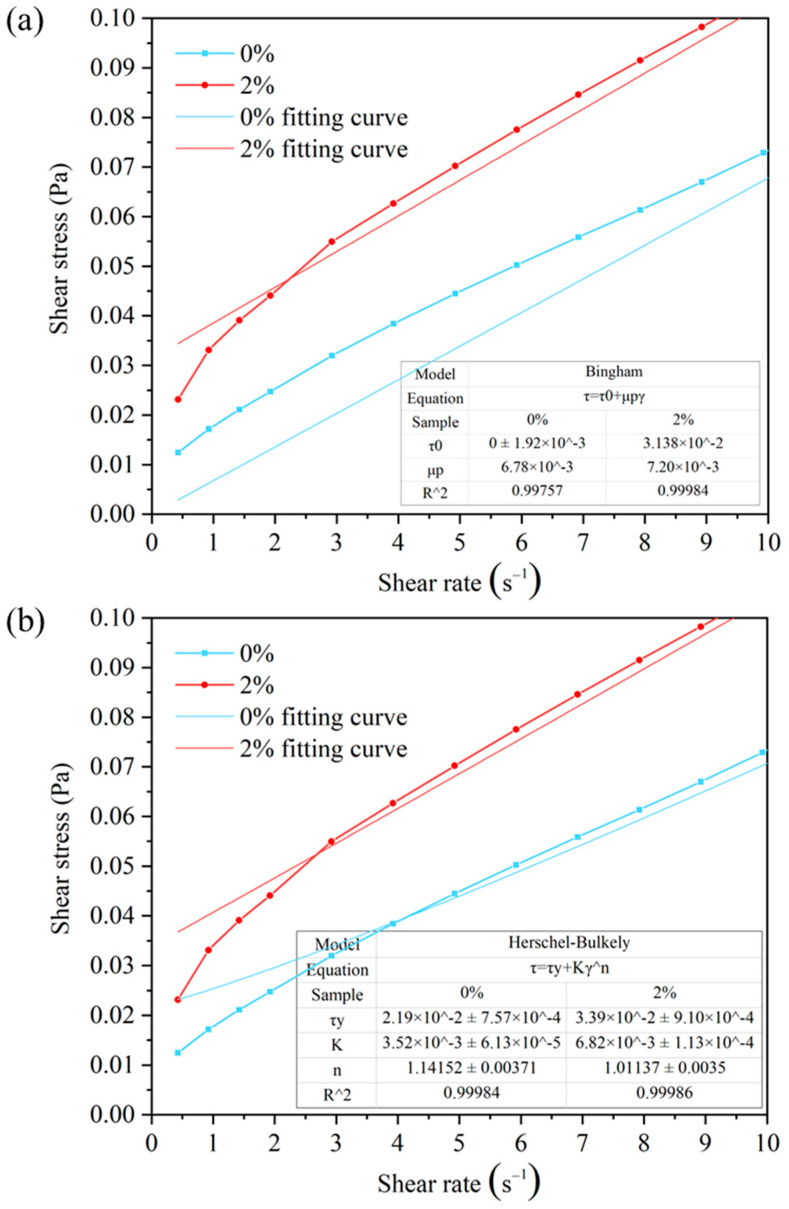
Rheological fitting model of drilling fluid ((**a**) Bingham model; (**b**) Herschel–Bulkley model).

In addition to studying the rheological properties of the drilling fluids mentioned above, the flow pattern of the drilling fluid with HAS was also analyzed, as shown in Figure 7. It was found that when fitting with the Bingham model, drilling fluid with 2% HAS was more in line with the Bingham model (Figure 7a). When fitting with the Herschel–Bulkley model, the drilling fluid with 2% HAS was more consistent (Figure 7b). However, in comparing their R^2^ values, the drilling fluid with 2% HAS was more in line with the Herschel–Bulkley model.

##### Performance Comparison Between HAS and Traditional Rheology Modifiers

**Figure 8:**
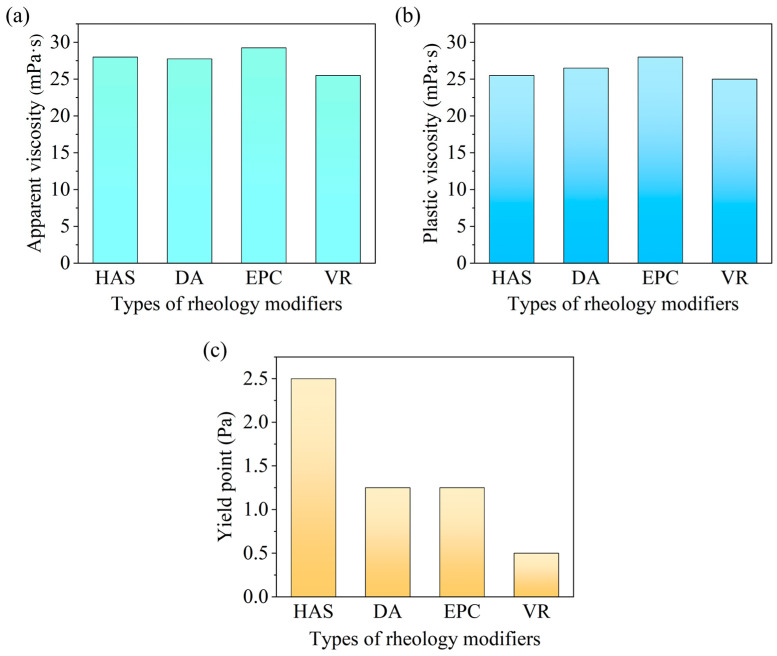
Performance comparison of different rheology modifiers ((**a**) apparent viscosity; (**b**) plastic viscosity; (**c**) yield point).

We selected three rheology modifiers—dimer acid (DA), ethylene–propylene copolymer (EPC), and vinyl resin (VR)—and their rheological properties were studied in comparison with HAS at −55 °C, as shown in Figure 8. The dosage was 2%.

This comparison indicated that among different rheology modifiers, drilling fluid with HAS exhibited the highest yield point, and its plastic viscosity was only 25.5 mPa·s. This suggested that the rheological performance of HAS surpassed that of the other three traditional rheology modifiers under ultra-low temperature conditions.

#### 2.2.2. Wetting Performance of HAS

##### Wettability of HAS in Drilling Fluids with Different Base Oil

**Figure 9:**
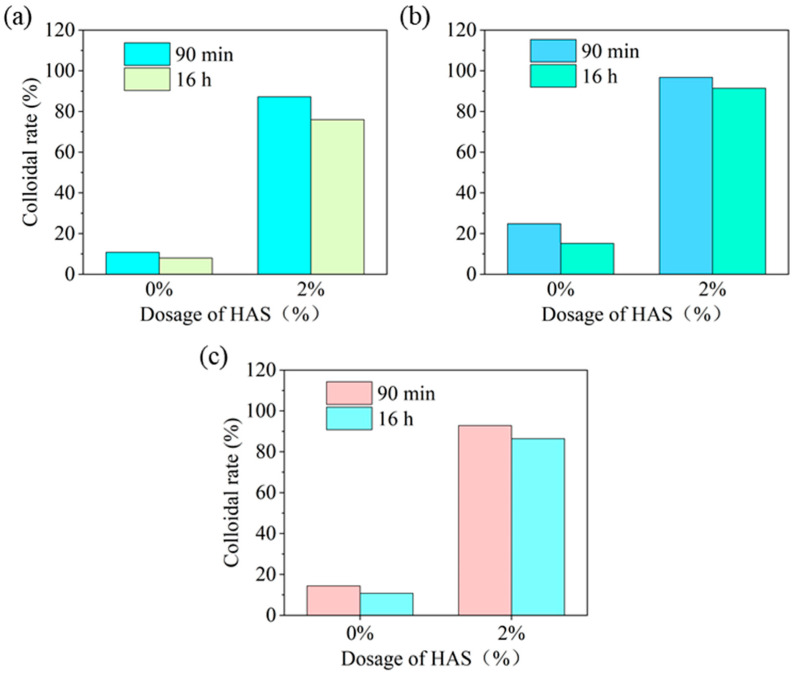
Colloid rate of HAS in different base oils ((**a**) aviation paraffin; (**b**) white oil; (**c**) mixed liquid).

Colloid ratio is an important standard to measure the dispersion stability of drilling fluid. The dispersion stability directly determines drilling fluid’s wettability. The excellent wettability of drilling fluid is an important feature of weak gel grid structure, and the colloid rate serves as a key method for assessing the wettability [17,18,19]. The influence of HAS in base oil on the colloid rate was studied at −55 °C (Figure 9).

When HAS was not added, the colloidal rate of organoclay (160F) was very low and the wettability was poor in different base oils. However, 2% HAS increased the colloidal rate to more than 76% in different base oils. This shows that HAS significantly enhanced the wettability for drilling fluid. This was mainly attributed to the ability of HAS to interact with organoclay in drilling fluid to form a network structure, which effectively improved the dispersion stability of drilling fluid.

##### Wettability of HAS in Drilling Fluid at Different Temperatures

**Figure 10:**
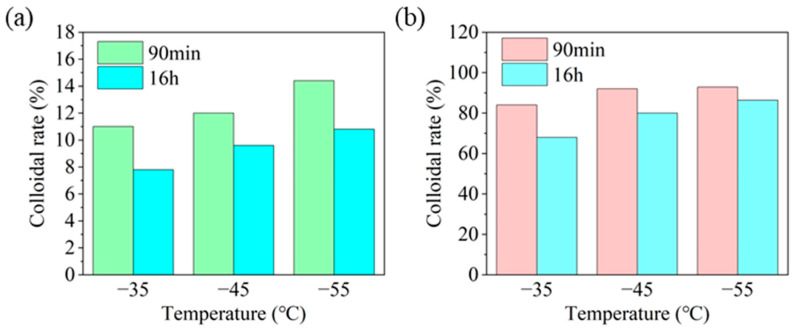
Effect of HAS on the colloid rate of drilling fluid at different temperatures ((**a**) 0%; (**b**) 2%).

Temperature also exhibits an obvious effect on the wettability for drilling fluid. As a result, the colloidal rate of drilling fluid with HAS was studied at different temperatures (Figure 10).

The range of colloidal rate (standing for 16 h) for drilling fluid was 7.8% to 10.8% (Figure 10a) at different temperatures, indicating poor wettability. Nevertheless, the colloid rate of drilling fluid with 2% HAS improved to 68~86.4% (Figure 10b). This indicated that HAS dramatically raised the wettability of drilling fluid at different temperatures.

##### Wettability of HAS in Drilling Fluid at Different Densities

When drilling in Antarctica, maintaining proper drilling fluid density is essential for ensuring safety and efficiency. The density range for Antarctic drilling fluid is 0.92~0.95 g/cm^3^. The colloidal rate was investigated by adding the HCFC-141b density modifier to the drilling fluid at different densities.

**Figure 11:**
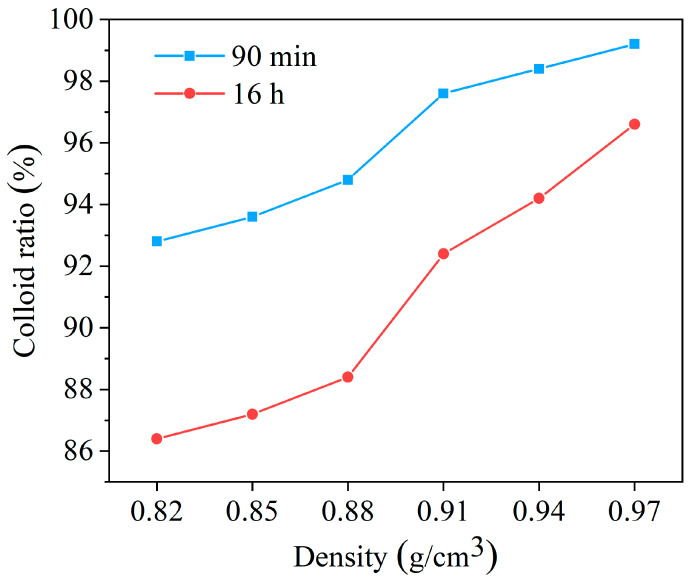
Effect of HAS on colloid rate for drilling fluid at different densities.

From Figure 11, as the density improved continuously, the colloid rate showed a gradually increasing trend. In addition, the colloid rate remained above 92.4% while the density was 0.92~0.95 g/cm^3^, indicating excellent colloid stability and good compatibility between HAS and drilling fluid under different density conditions.

##### Wettability of HAS in Drilling Fluids with Different Organoclays

In order to study HAS universality, the influence of HAS on colloid rate of drilling fluids with different organoclays was explored separately.

**Figure 12:**
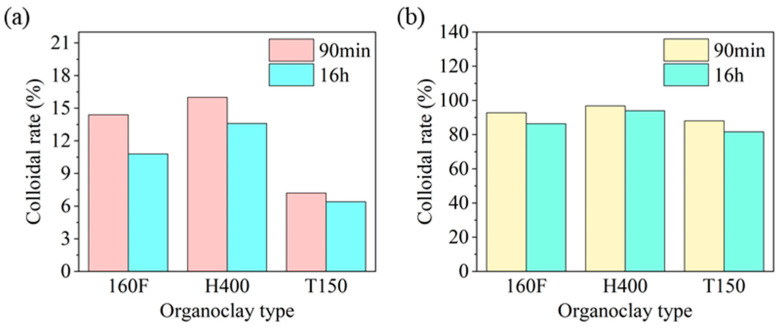
The effect of HAS on the colloid rate for drilling fluids with different organoclays ((**a**) 0%; (**b**) 2%).

By studying the colloidal rates at −55 °C, it was found that the three types of organoclays had very low colloidal rates before the addition of HAS, and the 160F organoclay had a colloidal rate of only 10.8% after standing for 16 h (Figure 12a). However, when HAS was added, the colloidal rates of the three organoclays were significantly improved (Figure 12b). The H400 organoclay had the highest colloidal rate (94%) and the T150 organoclay had the lowest (81.6%). This analysis indicated that when organoclay had large interlayer spacing, small particle size, and loose arrangement, it would exhibit excellent colloidal dispersibility in drilling fluid. This also better explained the mechanism by which HAS caused different organoclays to have different colloidal rates in drilling fluid.

#### 2.2.3. Assessment of Environmental Compatibility for HAS

As shown in Table 1, the experimental study indicated that the BOD_5_ was 8996.3 mg/L and COD was 28,936.0 mg/L. The calculated value of BOD_5_/COD was 31.1%. It showed that HAS was an environmentally friendly material.

#### 2.2.4. Filtration Performance for Drilling Fluid

Based on the aforementioned research, the filtration performance of the drilling fluid with HAS was further evaluated (Table 2). The study revealed that the API filtration loss was only 14 mL, and its mud cake surface was smooth, thin, and dense. This indicated that the drilling fluid exhibited good filtration performance.

Through research on the wettability, rheological properties, and filtration performance of drilling fluid, it was found that drilling fluid with HAS exhibited excellent wettability under ultra-low temperature conditions. Additionally, it also demonstrated good rheological and filtration properties. This was primarily due to the formation of a spatial network structure between multiple polar groups in HAS and organoclay particles through hydrogen bonding, which subsequently improved the wettability. Simultaneously, HAS effectively enhanced the rheological and filtration properties of the drilling fluid.

### 2.3. Mechanism Research

#### 2.3.1. Particles Size and Number

**Figure 13:**
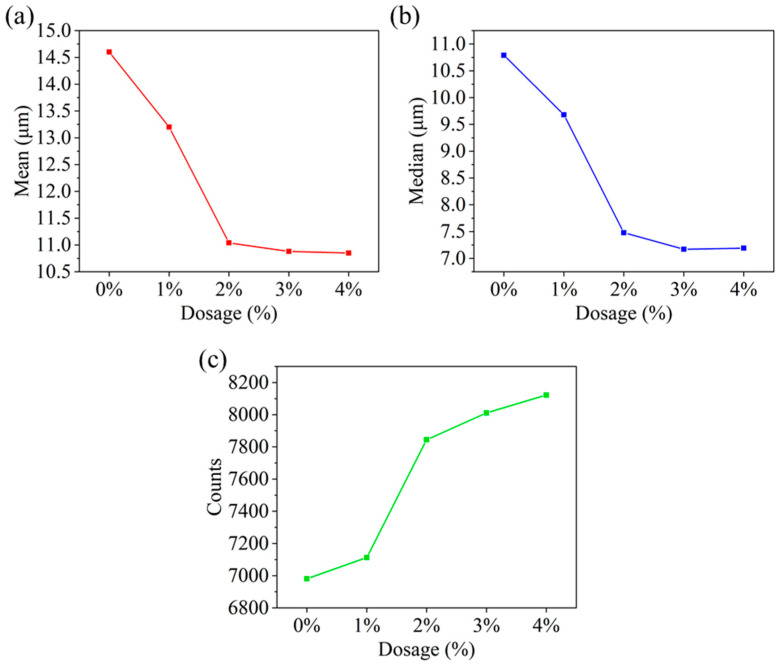
Effect of HAS on the number and size of particle of organoclay for drilling fluids ((**a**) particle mean; (**b**) particle median; (**c**) particle counts).

Through assessing the impact of HAS on the particle size and number of organoclay at −55 °C, its action mechanism was further investigated (Figure 13).

As the HAS dosage improved, the mean and median of the particles decreased continuously. Among them, 2% HAS reduced the particle mean from 14.6 μm to 11.04 μm, and the median from 10.79 μm to 7.48 μm. In addition, the particle counts increased from 6981 to 7844. It was shown that HAS interacted with the organoclay particle by forming hydrogen bonds, which in turn prevented particle aggregation, making an improvement in particle counts and a diminishment in particle size [20,21].

#### 2.3.2. Microscopic Morphology

**Figure 14:**
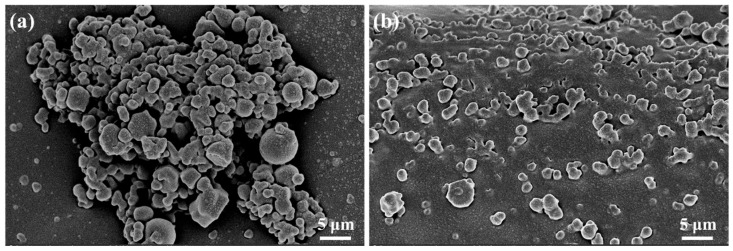
Effect of HAS on drilling fluid micromorphology ((**a**) 0%; (**b**) 2%).

The impact of HAS on the microscopic morphology of drilling fluid was explored by cryo-SEM (Figure 14).

It was found that when HAS was not added, the organoclay tightly accumulated in the drilling fluid, and the wettability was very poor (Figure 14a). However, HAS effectively enhanced the drilling fluid’s wettability (Figure 14b). Meanwhile, it could also be observed that HAS significantly reduced the particle size of organoclay, which was consistent with the results in Figure 9.

#### 2.3.3. Mechanism Analysis

**Figure 15:**
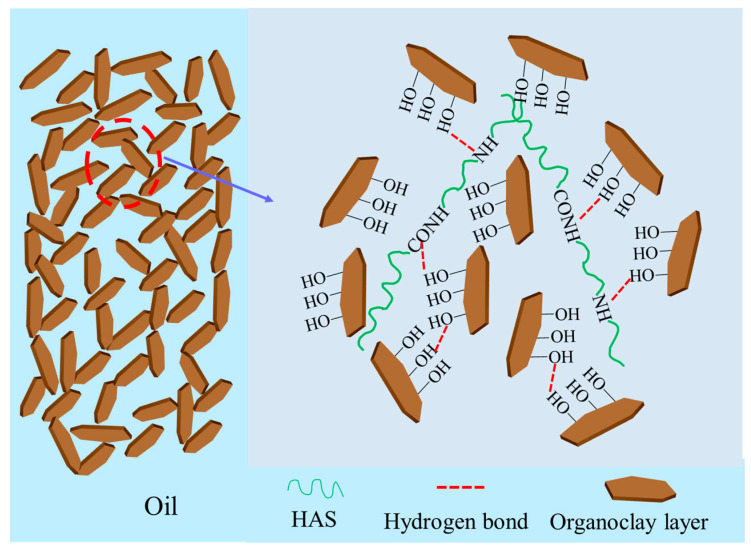
Schematic diagram of action mechanism for HAS.

To vividly describe the action mechanism for HAS in drilling fluid, a schematic diagram is shown into Figure 15. As shown in the figure, the HAS molecule has a lipophilic long carbon chain and polar groups such as amide and imine groups, which gives it an amphiphilic structure. The lipophilic long carbon chain makes it stretch in the base oil, while multiple polar groups enable it to form a weak gel grid structure with organoclays particle through hydrogen bonding. In addition, HAS can also form intermolecular forces through interactions such as intermolecular entanglement. The above combined effect enables organoclays particle to maintain long-term stability in base oil, thereby obviously enhancing the wettability and rheology of drilling fluid.

## 3. Conclusions

This research prepared an environmentally friendly material (HAS) that significantly ameliorated the wettability and rheology for drilling fluids at ultra-low temperature. The studies revealed that 2% HAS increased the colloid rate from 8.0% to more than 76.0% in different base oils at −55 °C. When the density was 0.92~0.95 g/cm^3^, the colloid rate remained above 92.4%. In addition, 2% HAS made the colloid rate of drilling fluid with H400 reach 94.0%. More notably, 2% HAS enhanced the yield point from 1.5 Pa to 2.5 Pa, and the plastic viscosity of drilling fluid was only 25.5 mPa·s at −55 °C, significantly improving the drilling fluid rheology. In addition, the drilling fluid with HAS maintained a filtration loss of 14 mL after cooling at −55 °C for 16 h, demonstrating good filtration performance. The above results indicated that the research objectives were achieved. Mechanistic studies indicated that HAS formed a weak gel grid structure through the interactions of polar groups such as amide and imino groups with organoclay particles, thus improving the rheology and wettability of the drilling fluid. More notably, HAS is an environmentally friendly material, which gives it broad application prospects in Antarctic drilling.

## 4. Materials and Methods

### 4.1. Materials

Dodecylamine polyoxyethylene ether (AR) and *N*-ethylethylenediamine (AR) were both purchased from Qingdao Zhongshi Boyuan Biotechnology Co., Ltd. (Qingdao, China), azelaic acid (AR) came from Qingdao Xinyu New Material Technology Co., Ltd. (Qingdao, China), 160F organoclay, H400 organoclay, and 120F organoclay were all provided by Shanghai Wanzhao Fine Chemical Co., Ltd. (Shanghai, China), QY white oil and aviation paraffin (No. 4) came from Shandong Luying Chemical Co., Ltd. (Jinan, China), concentrated sulfuric acid came from Qingdao Molin Trading Co., Ltd. (Qingdao, China), and HCFC-141b was provided by Shanghai Ruiyi Environmental Protection Technology Co., Ltd. (Shanghai, China).

### 4.2. HAS Preparation

A total of 30 g dodecylamine polyoxyethylene ether (AR, Mw: 273, oil-soluble) and 11.28 g azelaic acid (purity: 99%, Mw: 188.22) were introduced into the reaction vessel and mixed thoroughly by stirring for 10 to 15 min. Soon afterwards the above mixture was heated to 130 °C after introducing nitrogen for 20 min. Next, 0.83 g concentrated sulfuric acid (98%) was added into the mixture and it was reacted at 130 °C for 3 h to obtain the first reaction product. Then, 11.39 g *N*-ethyl ethylenediamine (98%) was added to the above mixture, and it was held at 175 °C for 4 h to acquire the HAS.

### 4.3. Characterization of HAS

#### 4.3.1. Infrared Spectral Analysis

Firstly, the HAS was prepared via the ATR method for the sample to be tested, and then the sample was characterized by a Hoffen-20 Fourier Transform Infrared (FTIR) spectroscopy scanner (Tianjin Jiaxinhai Mechanical Equipment Co., Ltd., Tianjin, China), with the parameters including the resolution (4 cm^−1^) and the wave number range (400~4000 cm^−1^).

#### 4.3.2. The Analysis of Nuclear Magnetic Hydrogen Spectrum

The HAS sample was first dissolved in deuterated chloroform, and then the H spectra were investigated by ^1^H-NMR (Bruker AV400 MHz, Berlin, Germany) to verify that the sample was prepared successfully.

### 4.4. Preparation of Base Fluid and Antarctic Drilling Fluid

The base oil was formulated by blending aviation paraffin (No. 4) and white oil (QY) in a 7:3 volume ratio. Subsequently, 3% organoclay (160F) was added to the base oil and stirred for 30 min to form the base fluid. Lastly, 2% HAS was added to the base fluid, and it was stirred for 20 min to prepare the Antarctic drilling fluid. The drilling fluid formula was as follows: 70% aviation kerosene + 30% white oil + 3% organoclay + 2% HAS.

### 4.5. Performance Evaluation

#### 4.5.1. Rheological Performance of HAS

To prepare the Antarctic drilling fluid, 2% HAS was added to the base fluid. The rheological parameters were evaluated using an ultra-low temperature viscometer at −55 °C. The formulas are as follows:

(1) AV=0.5Φ600

(2) PV=Φ600−Φ300

(3) YP=0.5×(2×Φ300−Φ600)

Shear dilution behavior: The sample was cooled at −55 °C for 16 h, and its viscosity variation with shear rate was tested by a Haake rheometer at 4 °C. The setting range of the shear rate was 0~100 s^−1^.

Thixotropy: The thixotropic loop area was tested by HAAKE rheometer. Firstly, the variation curve of shear stress with the increase in shear rate (0 to 200 s^−1^) was tested by controlling the program. Secondly, the shear stress variation curve obtained by decreasing shear rate (200 to 0 s^−1^). The thixotropy was evaluated by calculating the area enclosed by these two curves. The area of the thixotropic loop is automatically calculated by the HAAKE rheometer (Thermo Fisher Scientific, Waltham, MA, USA), and the unit is pa/s. The drilling fluid was cooled at −55 °C for 16 h and tested at 4 °C.

The relative magnitude of elastic modulus (G′) and viscous modulus (G″) can determine the viscoelastic characteristics of sample [22,23]. If G′ is higher than G″ within a specific range of shear stress, it is an elastic fluid; if it is contrary to this, it is a viscous fluid. The modulus variation with shear stress was measured via HAAKE rheometer. The drilling fluid was cooled at −55 °C for 16 h and tested at 4 °C.

#### 4.5.2. Wettability of HAS

The drilling fluid with HAS was agitated for 25~30 min by high-speed stirrer at a low-temperature. Then, the sample was loaded into a stoppered measuring cylinder and kept at this condition from 90 min to 16 h at a specific temperature. The wetting performance was studied using the following formula:

(4) $$Cr=\frac{V1}{V2}\times 100\%$$

where *Cr*—colloid rate, %;

*V*_1_—colloid volume, mL;

*V*_2_—total volume, mL.

#### 4.5.3. Environmental Compatibility Assessment

When conducting scientific expeditions in the Antarctic region, there are high requirements for the environmental friendliness. Hence, it is very essential to explore the eco-friendliness of HAS. Biodegradability tests were conducted in accordance with relevant standards [24]. The sample (HAS) biodegradability was studied by the specific value of BOD_5_ and COD.

#### 4.5.4. Evaluation of Filtration Performance

Firstly, the prepared drilling fluid was cooled at −55 °C for 16 h. Subsequently, the drilling fluid was poured into a measuring apparatus, and the filtration loss was measured using a medium-pressure filtration tester at 100 psi according to API standards.

### 4.6. Studies of Action Mechanism

#### 4.6.1. Particle Size and Number

Initially, the drilling fluid with HAS was stirred for 20 min at speed of 600 r/min. Secondly, the Particle Track G400 laser particle size analyzer (Mettler Toledo Technology Co., Ltd., Shanghai, China) was used to test the changes in particle size and number. During the experiment, an ultra-low temperature environment was achieved by cryogenic circulation pump. Each sample was tested five times, with averages reported as final values.

#### 4.6.2. Cryo-SEM

Initially, the drilling fluid with HAS was agitated for 25 min. Subsequently, it was cryogenically frozen (−160 °C) and subjected to a sublimation operation. Next, the sample was gold-sprayed. Finally, its microstructure was studied by NANOS scanning electron microscope (Shanghai Maike Inno Scientific Instruments Co., Ltd., Shanghai, China) [16,25].

## Figures and Tables

**Table 1:** Environmental indicator evaluation result.

Name	Measured Value	Standard Value	Classification
BOD_5_: COD, %	31.1	>25	Environmentally friendly

**Table 2:** Evaluation of drilling fluid filtration performance.

Drilling Fluid Formulation	Test Conditions	FL_API_ (mL)	Mud Cake Morphology
70% aviation kerosene + 30% white oil + 3% organoclay + 2% HAS	Cooling at −55 °C for 16 h	14 mL	thin and dense

## Data Availability

The original contributions presented in this study are included in the article. Further inquiries can be directed to the corresponding author.
